# Transcutaneous delivery of sodium bicarbonate increases intramuscular pH

**DOI:** 10.3389/fphys.2023.1142567

**Published:** 2023-03-07

**Authors:** Brandon M. Gibson, Karen Wiedenfeld Needham, Brendan W. Kaiser, Brad W. Wilkins, Christopher T. Minson, John R. Halliwill

**Affiliations:** Department of Human Physiology, University of Oregon, Eugene, OR, United States

**Keywords:** athletic performance, metabolic acidosis, acidosis lactic, anaerobic threshold, alkalosis

## Abstract

**Introduction:** Oral bicarbonate loading improves the buffering of metabolic acidosis and may improve exercise performance but can also result in gastric distress. Momentous’ PR Lotion contains a novel composition intended to provide a transdermal delivery vehicle for sodium bicarbonate which could allow the same ergogenic effect without the gastric distress. The present study explored the effect of transdermal delivery of sodium bicarbonate in a resting condition.

**Methods:** We measured the pH from intramuscular dialysate, *via* microdialysis, of the *vastus lateralis* during a 2 h application of PR Lotion (40 g of lotion per leg) in 9 subjects (3 women, 6 men). Venous blood samples were obtained for serum pH before and after application. A placebo time control was also performed in 4 subjects (2 women, 2 men). We hypothesized that PR Lotion application would increase pH of intramuscular dialysate.

**Results:** PR Lotion resulted in a rise in pH of 0.13 ± 0.04 units (*p* < 0.05), which translates to a 28% reduction in [H^+^]. Increases in serum pH were smaller (∼9%) yet consistent (*p* < 0.05). In contrast, placebo time control pH tended to decrease (*p* = 0.08). The effect of PR Lotion on pH tended to correlate with the dose per kg body weight of each individual (*r* = 0.70, *p =* 0.08).

**Conclusion:** These observations support the idea of transdermal bicarbonate delivery impacting pH buffering both systemically and intramuscularly. Further work investigating these potential benefits in an exercising model would be critical to establishing PR Lotion’s utility as an ergogenic aid.

## 1 Introduction

Exercise of a sufficient intensity, where energy demands must be met *via* non-mitochondrial ATP production, results in the localized accumulation of hydrogen ions (H^+^) and reduction in pH thought to disrupt glycolytic enzymes essential to exercise continuation (Trivedi and Danforth, 1966; Robergs et al., 2004). Termed “metabolic acidosis,” this is also thought to unfavorably affect the muscle microenvironment and contribute to the sensation of fatigue, characterized by a diminished force production despite increases in effort perception (Mainwood and Worsley Brown, 1975; Mainwood and Cechetto, 1980; Chase and Kushmerick, 1988; Swank and Robertson, 1989; Enoka and Stuart, 1992; Swank and Robertson, 2002). Further, acidosis is associated with altered potassium (K^+^) and sodium (Na^+^) concentrations in arterial and venous blood (Lindinger et al., 1999; Sostaric et al., 2006), in addition to muscle interstitium (Street et al., 2005). Disturbances in these ion concentrations have been implicated in depressed force production and muscle fatigue (McKenna et al., 2008).

For decades, the bicarbonate buffering system has been a target of ergogenic supplements and interventions, primarily using oral loading of exogenous sodium bicarbonate in the hours to days preceding an intense exercise bout (Galloway and Maughan, 1996; Marx et al., 2002; Carr et al., 2011; Calvo et al., 2021). While oral sodium bicarbonate loading increases blood pH and bicarbonate concentration and can result in improvements in exercise performance (Hirche et al., 1975; Kesl and Engen, 1998), it has often been associated with performance encumbering gastric distress when consumed at “ergogenic” doses of 0.3 g/kg to 0.5 g/kg body weight (Carr et al., 2011). Various strategies of oral loading have been employed to mitigate gastric distress, however, even when split doses are used deleterious side effects often persist (Oliveira et al., 2017).

Momentous’ PR Lotion contains a proprietary composition intended to provide a transdermal delivery vehicle for sodium bicarbonate that could improve bicarbonate buffering of H+ produced during exercise, without the potential for gastric distress. Initial work by McKay *et al.*, (McKay et al., 2020), compared the effects of PR Lotion to oral loading of sodium bicarbonate and found no changes in systemic acid-base status with PR Lotion, but did for oral sodium bicarbonate. However, the authors (McKay et al., 2020) acknowledged that without information on how well the topical bicarbonate permeates the multiple layers of skin, it is difficult to compare the actual dose of sodium bicarbonate delivered through oral consumption versus topical means.

Thus, the current study was designed to explore the effectiveness of PR Lotion as a transdermal delivery vehicle for sodium bicarbonate in a resting condition. We hypothesize that a rise in pH within intramuscular dialysate would be observed with a concomitant rise in serum pH following high dose PR Lotion application to the skin surface of the lower extremities during a resting condition. Secondarily, we hypothesized that the application of sodium in the sodium bicarbonate lotion and the corresponding alkalosis will contribute to an increase in circulating Na^+^ concentrations and reduced K^+^ concentrations within the intramuscular dialysate.

## 2 Materials and methods

### 2.1 Subjects

Nine individuals (3 women, 6 men) participated in the primary study and four individuals (2 women, 2 men) participated in a follow-up placebo control study. All subjects were healthy, non-smokers, and physically active (Table 1). Three subjects participated in both studies (1 woman, 2 men). All follow-up control studies were separated by a minimum of 6 weeks from the primary study completion. Subject characteristics were obtained during an initial screening visit for age, body mass, and height. Body surface area and body mass index were calculated from mass and height. Subjects did not use prescription medications (apart from oral contraceptives), performance enhancing drugs, or dietary supplements that may have antioxidant or recovery altering properties as determined by a self-reported health screening questionnaire.

**TABLE 1:** Subject characteristics.

Characteristic	Primary study	Placebo control study
*N*	9 (3W, 6M)	4 (2W, 2M)
Age, y	30 ± 8	28 ± 3
Height, cm	175 ± 10	172 ± 9
Weight, kg	72.9 ± 16.3	75.2 ± 20.2
Body mass index, kg/m^2^	23.6 ± 3.6	24.8 ± 4.5
Body surface area, m^2^	1.87 ± 0.24	1.88 ± 0.29

Values are expressed as means ± SD. W, women; M, men.

All studies were approved by the Institutional Review Board at the University of Oregon and were performed in accordance with the principles outlined by the Declaration of Helsinki. Written informed consent was obtained from all subjects after a verbal and written briefing of all experimental procedures.

### 2.2 Study design

All study visits were held in the morning hours and subjects were asked to arrive to the laboratory wearing typical exercise clothing, shorts and a t-shirt, having abstained from food for the 2 h preceding testing, caffeine for 6 h, alcohol for 12 h, and strenuous exercise for 24 h. Upon arrival at the laboratory, a urine sample was collected to screen for pregnancy (if applicable). Subjects were instrumented in the supine position with an antecubital intravenous catheter for blood sampling, 3-lead electrocardiogram, and intramuscular microdialysis probe in the *vastus lateralis* for interstitial sampling. Once the probe was placed, 90 min was allowed for resolution of placement trauma prior to collection of baseline samples for blood and dialysate. Room temperature remained thermoneutral (∼23°C) throughout the study.

Following baseline sampling, in the primary study, 40 g of PR Lotion (Momentous, Park City, UT, United States) was applied by an investigative team member over the entire surface area of each leg from ankle to groin. As PR Lotion is 33% sodium bicarbonate, a total of 26.4 g of sodium bicarbonate were applied. For the follow-up placebo control study, 40 g of a placebo version of PR Lotion, which included all the ingredients of PR Lotion except for sodium bicarbonate, was applied to each leg.

**FIGURE 1:**
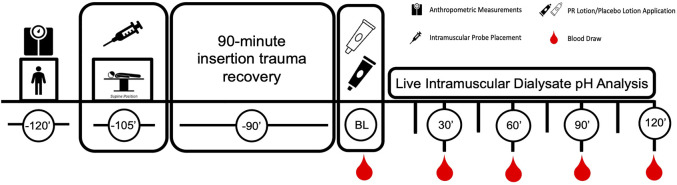
Study Design.

Throughout the studies, microdialysate samples were analyzed in real time with a flow-through ion-selective microelectrode for pH and dialysate was collected over 20 min intervals for later off-line analysis of K^+^ and Na^+^ concentrations. Blood samples were collected immediately preceding PR Lotion application baseline (0 min) and at 30, 60, 90, and 120 min after lotion application (Figure 1).

#### 2.2.1 Intramuscular microdialysis

An intramuscular microdialysis probe was placed into the *vastus lateralis* using sterile technique and a splittable introducer so that it was parallel to the pennation angle of the muscle fibers (Romero et al., 2017). The skin was initially anesthetized using prilocaine hydrochloride (4%) (Citanest^®^, Septodont, Lancaster, PA, United States) followed by a lidocaine (2%) and epinephrine (1:100,000) solution (Lignospan^®^, Septodont, Lancaster, PA, United States) applied down to the underlying fascia. Care was taken to ensure that prilocaine and lidocaine were not injected into the skeletal muscle. After insertion, probes were held in place by covering the entry site with a sterile transparent medical dressing. The probe had a 20-kDa molecular mass cutoff with a 30-mm polyarylethersulfone membrane (63 MD Catheter, MDialysis, Stockholm, Sweden). The semipermeable membrane chosen in this probe allows H^+^_,_ K^+^, and Na^+^ to freely diffuse from the interstitial space, providing quantifiable measures of the ability of PR Lotion to deliver molecules to the skeletal muscle tissue and affect change in the interstitial environment. Following insertion, the microdialysis probe was perfused continuously at a rate of 5 μL/min (CMA 400 Microdialysis pump, CMA, North Chelmsford, MA, United States) with a 0.9% saline solution. The distal end of the probe was connected to a custom low-volume (3 µL) flow-through manifold housing ion-selective pH and reference microelectrodes (16-705/16-702, Microelectrodes, Bedford, NH, United States) recording to an isolated multifunction data acquisition system (EPU452, eDAQ, Colorado Springs, CO, USA). After passing the manifold, dialysate was collected in microtubes then stored at −80°C for later analysis of K^+^ and Na^+^ concentrations.

#### 2.2.2 Blood measurements

Venous whole blood samples were collected into 5 mL Vacutainer^®^ tubes (Becton Dickinson, Franklin Lakes, NJ, United States) containing no anti-clotting agents. Samples clotted for 30 min at room temperature before being centrifuged (10 min at 1,300 relative centrifugal force) and separated. Serum was analyzed for pH (Φ™ 200 Series, Beckman Coulter, Indianapolis, IN, United States) and stored at −80°C for later analysis of K^+^ and Na^+^ concentrations.

#### 2.2.3 Biochemical analysis

Concentrations of K^+^ and Na^+^ were measured in dialysate and serum *via* flame photometry (BWB Technologies United States, Evans Mill, NY, United States) per manufacturer’s instructions. To eliminate intersample variance, all samples were thawed once and analyzed in the same run by a single technician. Sample results were inspected for visual stability across the sample run time and all samples with reported instability were re-processed. Sample results were recorded in millimolar (mM) concentrations in duplicate.

### 2.3 Statistical analysis

When measuring ion concentrations in small volumes of fluid, occasional values appear outside the normative range of value and may reflect artifactual measurements. These outliers can have an oversized impact on group means when the number of participants is small. As such, we used a standard criterion of z-scores of ±3 to identify and exclude outliers prior to deriving group means and standard errors (SE) or completing statistical analysis. A total of 594 data points were collected across 13 subjects and, of those, 7 were identified as outliers and excluded.

Primary and secondary outcome variables were analyzed with a mixed linear regression model to determine time course effects and presented as a line of best fit and upper and lower confidence limits for the regression. In addition, change (Δ) from baseline to end-of-study for each outcome variable was tested by univariate *t*-test. Lastly, to explore the possibility of a dose-pH relation, the absolute dosage of 80 g PR Lotion applied, made relative to participant’s bodyweight, was compared to the reported change in pH of the intramuscular dialysate *via* Pearson correlation. All variables are presented as mean ± standard error of the mean (SE). Significance was accepted at *p* < 0.05.

## 3 Results

### 3.1 Primary outcome variable: pH

**FIGURE 2:**
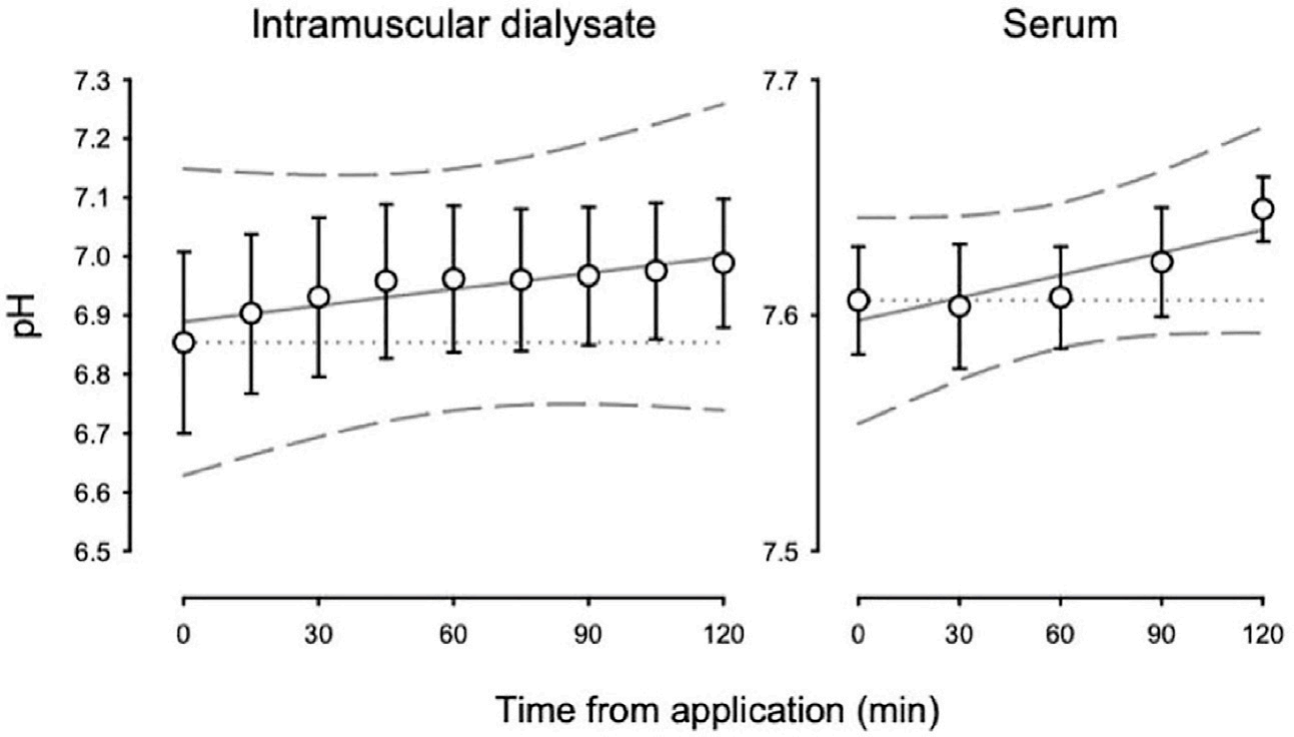
Intramuscular dialysate pH (left panel) and serum pH (right panel) during the primary study from baseline to end-of-study.

Figure 2 displays the pH for intramuscular dialysate and serum from the primary study at baseline, prior to application of the lotion, through 120 min following application. For intramuscular dialysate, pH increased at 0.058 ± 0.016 units per hour (*p* < 0.05). Likewise, for serum, pH increased at 0.019 ± 0.008 units per hour (*p* < 0.05). Table 2 shows the change in pH from baseline to end-of-study. Intramuscular dialysate and serum pH both increased (*p* < 0.05) over the duration of the study. In contrast, intramuscular dialysate pH tended to decrease over the course of the placebo time control study (Table 2).

**TABLE 2:** *Change in pH, Na+, and K+ from baseline to end-of-study*.

	Primary study		Placebo control study	
pH	Δ	*p*	Δ	*p*
Intramuscular	0.13 ± 0.04	<0.05	−0.24 ± 0.09	0.08
Serum	0.04 ± 0.01	<0.05	0.02 ± 0.01	0.22
[Na+]	Δ, mM	*p*	Δ, mM	*p*
Intramuscular	−1 ± 1	0.42	10 ± 1	<0.05
Serum	14 ± 5	<0.05	−3 ± 1	0.06
[K+]	Δ, mM	*p*	Δ, mM	*p*
Intramuscular	−0.32 ± 0.11	<0.05	−0.37 ± 0.07	<0.05
Serum	−0.01 ± 0.13	0.92	−0.11 ± 0.12	0.42

Values are expressed as means ± SE. *n* = 9 primary study, *n* = 4 placebo control study. *P* for univariate *t*-test for the change within a study.

### 3.2 Secondary outcome variables: K^+^ and Na^+^ concentrations

**FIGURE 3:**
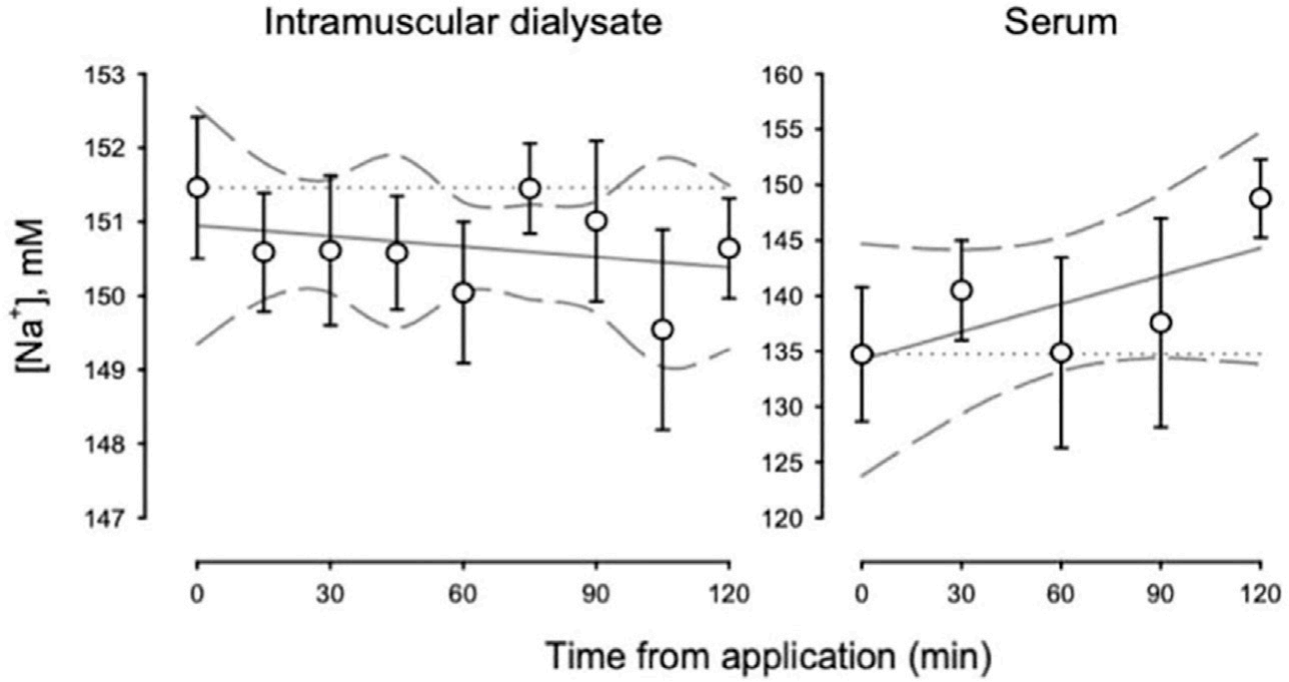
[Na^+^] for intramuscular dialysate (left panel) and serum (right panel) during the primary study from baseline to end-of-study.

Figure 3 shows the [Na^+^] for intramuscular dialysate and serum during the primary study from baseline, prior to lotion application, through 120 min following application. There were no clear trends for [Na^+^] to change across time in intramuscular dialysate or serum (*p >* 0.05). However, serum [Na^+^] did increase from baseline to end-of-study (*p* < 0.05; Table 2). For the placebo time control, we observed increases in [Na^+^] by end-of-study for intramuscular dialysate (*p* < 0.05) with a tendency for serum [Na+] to decline (Table 2).

**FIGURE 4:**
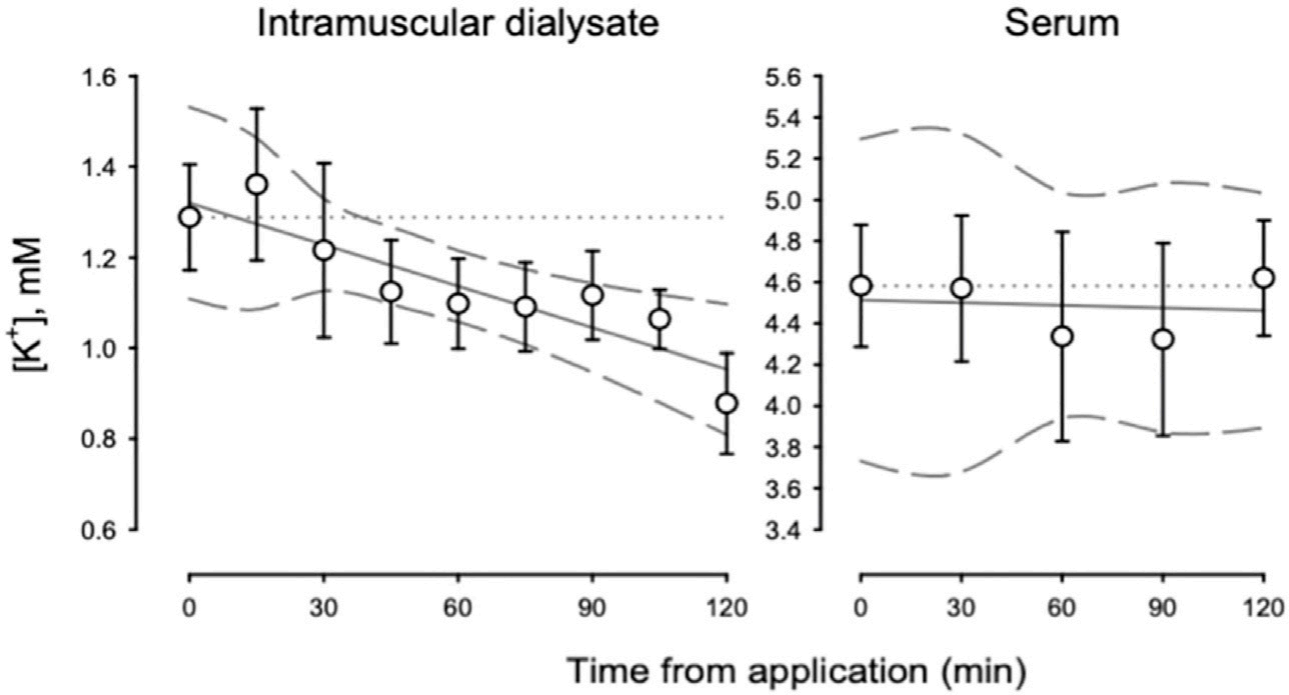
[K^+^] for intramuscular dialysate (left panel) and serum (right panel) during the primary study from baseline to end-of-study.

Figure 4 shows the [K^+^] for intramuscular dialysate and serum from the primary study from baseline prior to application through 120 min of application. Intramuscular dialysate, [K^+^] decreased at 0.126 ± 0.052 mM per hour (*p* < 0.05). Serum [K^+^] did not change over time. However, for the placebo time control, we observed a decrease in intramuscular dialysate [K^+^] by end-of-study (Table 2).

**FIGURE 5:**
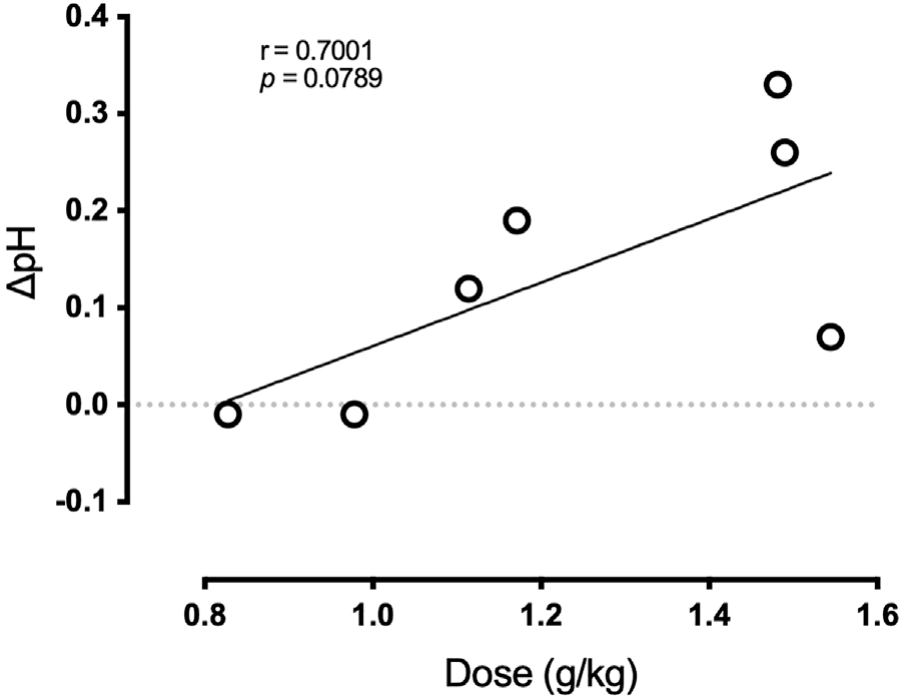
Relation of dosage to the change in intramuscular dialysate pH during the primary study.

Figure 5 displays the relation of dosage to the change in intramuscular dialysate pH from baseline to end-of-study. There was a trend for the change in pH to be greater in subjects who received higher doses relative to body mass of PR Lotion (*r* = 0.70, *p =* 0.080).

## 4 Discussion

The current study was designed to explore the effectiveness of PR Lotion as a transdermal delivery vehicle for sodium bicarbonate to skeletal muscle under a resting condition. According to the manufacturer, PR Lotion’s ingredients encapsulate hydrophilic molecules (e.g., sodium bicarbonate) for paracellular delivery, penetrate the stratum corneum, disrupt the junctional proteins of the skin barrier, and transiently create a paracellular pathway for the local delivery of molecular substances into the underlying tissues. While we did not directly test these claims, consistent with our hypothesis, the increase in pH of the intramuscular dialysate and serum are in support of PR Lotion’s proposed function. Indeed, the 0.13 increase in pH in dialysate from a baseline of 6.85 translates to a ∼28% reduction in [H^+^]. Changes in serum pH were smaller (∼9% reduction in [H+]) yet consistent with the anticipated alkalotic effect of PR Lotion application, increasing pH.

Taken together, our observations support the idea that transdermal bicarbonate delivery may impact pH buffering both intramuscularly and systemically. In contrast to the observed changes in pH, alterations in [Na^+^] and [K^+^] in both intramuscular dialysate and serum were less clear. With PR Lotion application, serum [Na^+^] was significantly elevated (Table 2) and tended to decrease in the placebo condition. However, other changes in Na^+^ and K^+^ concentrations were either inconsistent or were observed during both the primary study and the placebo time control (Table 2).

### 4.1 Bicarbonate loading

Early work by Costill *et al.*, (Costill et al., 1984), found that oral sodium bicarbonate could reduce metabolic acidosis in response to repeated bouts of supramaximal cycling and this was associated with improvements in cycling performance. Since then, the use of oral sodium bicarbonate as an ergogenic aid has produced mixed results due in part to differences in dosing practices, exercise models, acute ingestion versus chronic loading, and variation in gastrointestinal tolerance (Carr et al., 2011; Calvo et al., 2021).

A transdermal route of delivery, as proposed with application of PR Lotion, may be one approach to mitigate the negative consequences of gastrointestinal intolerance in the context of exercise performance. Along these lines, McKay *et. al,* (McKay et al., 2020), sought to compare the effectiveness of PR Lotion versus traditional oral ingestion of sodium bicarbonate. They found an increased blood pH with traditional oral administration, but not with PR Lotion application. It is worth noting that they only reported blood pH through 90 min following either lotion application or sodium bicarbonate ingestion. Our data suggest blood pH may not measurably change before 90 min, whereas we observed the highest serum pH to be evident at 120 min after lotion application. In fact, we observed a decrease in H^+^ concentration within skeletal muscle tissue of ∼28% with application of PR Lotion in resting subjects as well as a ∼9% reduction in serum H^+^ concentration at the end of the study period (120 min). Thus, timing of application may be important to observe expected effects.

Importantly, alkalosis in the serum and intramuscular dialysate was not observed in the placebo time control condition. Further, it is worth noting McKay et al., (McKay et al., 2020), used a PR Lotion dose of 0.90 g/kg of body weight, whereas the current study used, on average, 1.23 ± 0.28 g/kg. We also observed a trend for the change in pH to be related to PR Lotion dose (Figure 5; *p* = 0.08) and our results suggest that pH is more likely to be impacted when application dosage exceeds ∼1.0 g/kg. Therefore, a dosing strategy for PR Lotion that is relative to body weight may be more impactful.

### 4.2 Exercise performance and pH

The development of an acidic muscle environment in response to exercise has been shown in *ex vivo* animal models to reduce contractile function of skeletal muscle fibers where H^+^ are thought to competitively inhibit calcium binding at troponin-c, a specific regulatory site that allows for cross-bridge cycling to occur (Brenner, 1988; Parsons et al., 1997; Brenner et al., 1999). These findings are supportive of an improved pH status, like that observed in the present study, or a delay in the accumulation of H^+^, may be permissive to improvements in exercise performance through a maintenance of a more favorable environment essential for continued cross-bridge cycling. McKay et al., (McKay et al., 2020), explored this concept by comparing PR Lotion to a placebo but did not report any differences in performance during a time to exhaustion test or during a fixed performance task. However, they were also not able to show any difference between PR Lotion and traditional oral sodium bicarbonate on pH during these tasks. We speculate that the lack of an observable effect in this prior study may be due to application of a lower PR Lotion dose. Further work that explores the discrete impact of dose on exercise performance is critical to revealing the ergogenic utility of PR Lotion. Thus, cautious interpretation of the present findings, in the context of exercise performance, is warranted as no direct assessments on exercise performance were made in the present design.

### 4.3 Sodium loading

Kahle *et al.* (Kahle et al., 2013), suggested that a 70 kg athlete may consume on the order of 5700 mg Na^+^ with ergogenic doses of oral sodium bicarbonate. This quantity of sodium far exceeds the Dietary Reference Intakes Tolerable Upper Intake Level of 2,300 mg/d (Campbell, 2004). It is also above the range of consumption previously shown to increase mean arterial and systolic blood pressures when consumed for multiple weeks (Melander et al., 2007). Similar findings following 10 days of high sodium (6,200 Na^+^ mg/day) intake revealed augmented blood pressures during submaximal exercise potentially mediated by an impaired endothelial function (Babcock et al., 2020). Thus, the possibility exists that sodium bicarbonate loading may place some individuals at risk for the development of hypertension, particularly as sodium sensitivity increases with age (Weinberger and Fineberg, 1991), and benefits may be further limited by associated peripheral vasculature function impairment. While we did not observe changes in intramuscular dialysate Na^+^ concentrations with PR Lotion application, the serum increase of 14 ± 5 mM translates to a circulating sodium load of 800–900 mg (assuming a plasma volume 2.4–2.8 L). This appears to be greater than what has been reported with acute and chronic oral sodium bicarbonate loading of 0.5 g/kg body weight (Mc Naughton and Thompson, 2001). The current findings provide an appreciation of the efficacy of transdermal ion delivery in general, and highlight the potential risks associated with sodium bicarbonate loading irrespective of delivery method.

### 4.4 Strong ion and pH interactions

Muscle activation is critically dependent upon the Na^+^/K^+^ pump to re-establish resting ion gradients. An eventual dysregulation of gradients is thought to contribute to sensations of fatigue and a diminishment in muscle force production (Sreter and Woo, 1963; Juel, 1986; Nielsen and Overgaard, 1996). Overgaard *et al.* (Overgaard et al., 1999) highlighted the importance of assessing changes in both Na^+^ and K^+^ in combination rather than separately when resting gradients are lost in fatigue models. In the current study, the rise of Na^+^ concentration in the blood and reduction in K^+^ concentration in the intramuscular dialysate may suggest a shift to a greater amount of Na^+^ located extracellularly with a greater amount of K^+^ intracellularly. This could occur in response to an improvement in pH-sensitive Na^+^/K^+^ pump kinetics where greater activity has been reported in a more alkaline environment (Skou and Esmann, 1980). Similar reductions in K^+^ concentrations observed in the placebo time control suggests changes may be in response to influences beyond alkalinization caused by PR Lotion, such as fluid compartment shifts or other factors, complicating our results. Nevertheless, alterations in the ion concentrations of Na^+^ and K^+^ are differentially expressed across these physiological mechanisms and between the conditions, warranting further investigation.

### 4.5 Challenges of microdialysis

Intramuscular microdialysis provides unique insights into the intramuscular interstitium. It is ideally suited to tracking changes over time in small molecules such as those in the present study. That said, there are challenges inherent in the methodology in that one must balance flow rates with recovery rates to optimize quantification of solutes in the dialysate. As such, the dialysate concentrations that are detected will reflect patterns of change in interstitial concentrations over time, but they are not equal to the interstitial concentration. Small variations in probe manufacturer and larger variations in anatomy will result in a discrepancy in values of absolute pH as measured versus what it is within the tissue. Fortunately, these factors remain consistent within an experiment, but are the root cause of the large differences in baseline pH observed across subjects. For this reason, we place a greater emphasis on the change in pH than on absolute values. The change in pH in the present study is consistent with the rise observed in earlier work by Costill *et al.*, (Costill et al., 1984), reporting skeletal muscle pH after maximal exercise was preserved at a higher value (pH = 6.86 ± 0.06) in the sodium bicarbonate group compared to an exercising control (pH = 6.72 ± 0.04). As such we believe these changes are of a physiologically relevant magnitude to have a potential ergogenic effect.

### 4.6 Conclusion

This study was intended to explore the effectiveness of PR Lotion’s proprietary composition to act as a transdermal delivery vehicle for sodium bicarbonate. While we did not study subjects during exercise, the effects of PR Lotion on pH, Na^+^, and K^+^ in a resting condition suggest a potential benefit to exercise performance following application. Serendipitously, we may have revealed a minimal dosage requirement for alkalinization of skeletal muscle with the current formulation of Momentous’ PR Lotion. We note that changes in the concentrations of Na^+^ and K^+^ were contrary to what we expected (based on prior work), which complicates interpretation of these secondary outcomes, yet suggests PR Lotion is capable of altering ion concentrations at rest. Taken together, the observed increase in pH suggests a more favorable environment for maintained muscle excitability and force production, both key determinants of exercise performance. Further work investigating these potential benefits in an exercising model would be critical to determining PR Lotion’s utility as an ergogenic aid, as well as identifying its physiological impact on the muscle microenvironment during exercise that generates metabolic acidosis.

## Data Availability

The raw data supporting the conclusion of this article will be made available by the authors, without undue reservation.
